# Sterile Abscess in the Myocardium after Direct Intramyocardial Injection Related to Gene Therapy in a Swine Model

**DOI:** 10.5402/2011/319453

**Published:** 2011-04-06

**Authors:** Kiyotake Ishikawa, Dennis Ladage, Lisa Tilemann, Yoshiaki Kawase, Roger J. Hajjar

**Affiliations:** Cardiovascular Research Center, Mount Sinai School of Medicine, One Gustave L. Levy Place, P.O. Box 1030, New York, NY 10029-6574, USA

## Abstract

Cardiac gene therapy is one of the most promising approaches to cure patients with cardiac dysfunctions. Many ways of efficient gene transfer using viral vectors are tested, and some of them are already used in clinical settings. However, it is always important to be keenly alert to the possible complications when a new therapy is introduced. We present a case of myocardial sterile abscess in a swine model associated with a direct myocardial injection.

Research in cardiac gene therapy (CGT) is rapidly accumulating significant amounts of data. Many methods of gene delivery are currently being tested, and there are already several known ways to achieve sufficient gene transfer [1]. One of the most frequently used methods is direct intramyocardial injection of a viral vector [2, 3]. We present a case of myocardial sterile abscess associated with a direct injection of saline into the surrounding area of a myocardial infarction. 

The animal has received humane care in compliance with the Guide for the Care and Use of Laboratory Animals. A 20 kg Yorkshire pig was included in the control group of a CGT study using a myocardial infarction model. One week after an embolic coil implantation in the left anterior descending coronary artery, the pig underwent a direct intramyocardial injection of saline via a left lateral thoracotomy. First, access to the thoracic cavity was gained through the fourth intercostal space under the isoflurane anesthesia, the pericardial sac was cut, and the infarct area was then positively identified. A total of 500 *μ*L of saline was injected into 10 different sites along the border of the infarcted myocardium. After confirmation of hemo-stasis, the chest was closed and the pig was recovered. After recovery, the pig was cage-housed and examined daily for any signs of discomfort. No specific symptoms were observed and the pig gained weight at the same pace as the other pigs. However, 5 weeks after the thoracotomy, the pig was found dead in the cage just after the morning feeding. 

The heart was explanted and cut into 6 layers. An intramyocardial abscess was observed inside the infarct tissue along the anterior wall (Figure 1), however both tissue culture and Gram staining did not present any bacterial infection. Hematoxylin & Eosin staining showed significant amount of collagen tissue surrounded by acute inflammatory cells (Figure 2).

Myocardial abscess is a relatively uncommon disease which sometimes occurs as a complication of infective endocarditis. However, to our knowledge, this is the first report of sterile abscess in the myocardium. Previous studies have reported sterile abscess in different organs, mainly after the injection of vaccine [4] or hormones, and also after an implantation of biodegradable devices. Although saline was used for injection, our case presented the sterile abscess formation. Considering the fact that vaccination is one of the most frequent cause for sterile abscess formation, direct injection of viral vector into myocardium may raise the chance of this complication.

The result of H&E staining indicates that the sterile abscess formation is due to a significant inflammatory reaction. Open chest surgery and a relatively close period between the myocardial infarction and saline injection may have attributed to an increase in the reaction. 

Myocardial abscess has a certain resistance to medical therapy, and thus makes it difficult to treat the disease successfully. Although surgical intervention is the cornerstone of treating abscess, open drainage of the heart is challenging. In addition, patients with severe illness, who requires CGT, may have a greater risk of complications related to both anesthesia and surgery. Meanwhile, the chance of sudden death remains, and thus a poor outcome is expected with this disease.

 In conclusion, CGT using the direct intramyocardial injection method is a promising approach that can provide sufficient gene transfer. However, it is important to recognize this critical complication, and optimal treatment for this needs to be explored.

## Figures and Tables

**Figure 1:**
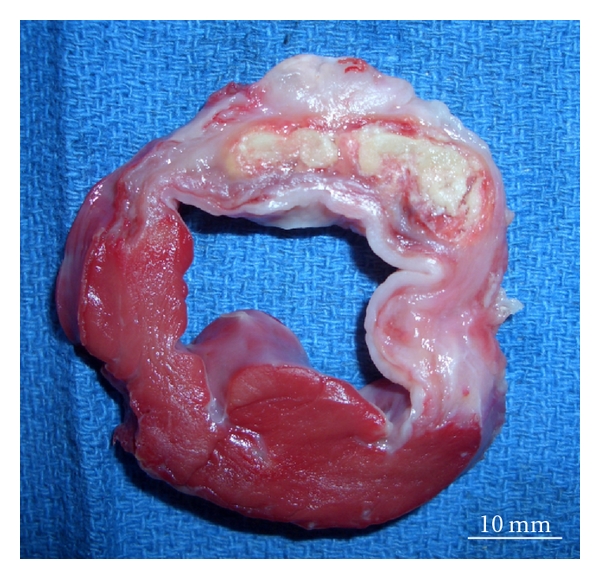
Cross-section of the heart at the papillary muscle level. The heart was stained by TTC to distinguish the infarct tissue from the normal myocardium. Intramyocardial abscess was detected inside of the infarct tissue. TTC = triphenyltetrazolium chloride.

**Figure 2:**
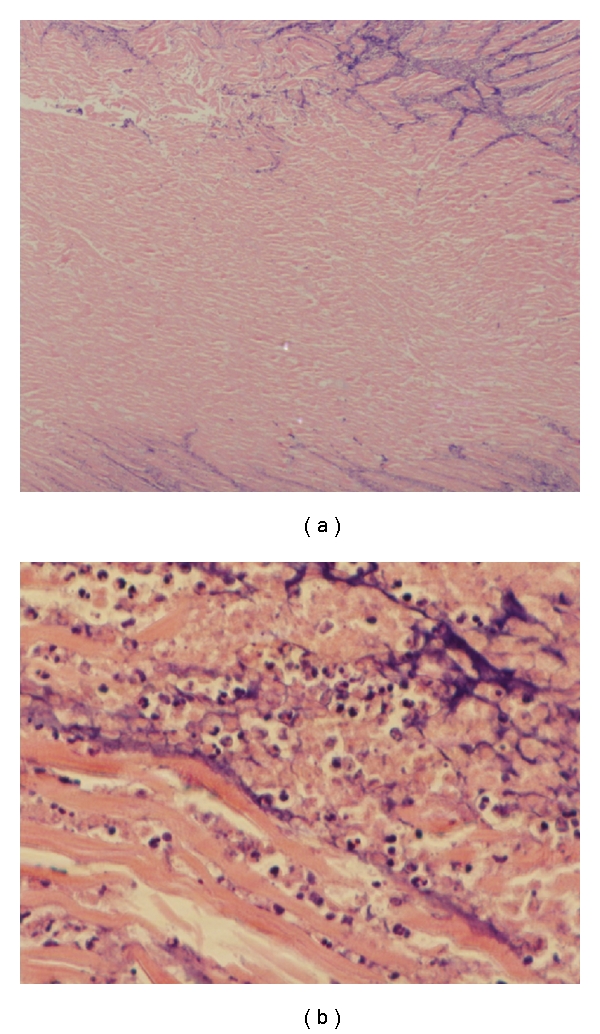
(a) Significant amount of fibrocollagenous tissue was surrounded by granulocytic infiltrates (H&E ×40). (b) Large numbers of reactive granulocytes, lymphocytes, and plasma cells are found in the surrounding area (H&E ×400). H&E = Hematoxylin & Eosin staining.
